# Receptoral Mechanisms for Fast Cholinergic Transmission in Direction-Selective Retinal Circuitry

**DOI:** 10.3389/fncel.2020.604163

**Published:** 2020-11-26

**Authors:** Joseph Pottackal, Joshua H. Singer, Jonathan B. Demb

**Affiliations:** ^1^Interdepartmental Neuroscience Program, Yale University, New Haven, CT, United States; ^2^Department of Biology, University of Maryland, College Park, MD, United States; ^3^Department of Ophthalmology and Visual Science, Yale University, New Haven, CT, United States; ^4^Department of Cellular and Molecular Physiology, Yale University, New Haven, CT, United States; ^5^Department of Neuroscience, Yale University, New Haven, CT, United States

**Keywords:** acetylcholine, direction selectivity, GABA, neural circuits, optogenetics, paracrine transmission, retina, synaptic transmission

## Abstract

Direction selectivity represents an elementary sensory computation that can be related to underlying synaptic mechanisms. In mammalian retina, direction-selective ganglion cells (DSGCs) respond strongly to visual motion in a “preferred” direction and weakly to motion in the opposite, “null” direction. The DS mechanism depends on starburst amacrine cells (SACs), which provide null direction-tuned GABAergic inhibition and untuned cholinergic excitation to DSGCs. GABAergic inhibition depends on conventional synaptic transmission, whereas cholinergic excitation apparently depends on paracrine (i.e., non-synaptic) transmission. Despite its paracrine mode of transmission, cholinergic excitation is more transient than GABAergic inhibition, yielding a temporal difference that contributes essentially to the DS computation. To isolate synaptic mechanisms that generate the distinct temporal properties of cholinergic and GABAergic transmission from SACs to DSGCs, we optogenetically stimulated SACs while recording postsynaptic currents (PSCs) from DSGCs in mouse retina. Direct recordings from channelrhodopsin-2-expressing (ChR2^+^) SACs during quasi-white noise (WN) (0-30 Hz) photostimulation demonstrated precise, graded optogenetic control of SAC membrane current and potential. Linear systems analysis of ChR2-evoked PSCs recorded in DSGCs revealed cholinergic transmission to be faster than GABAergic transmission. A deconvolution-based analysis showed that distinct postsynaptic receptor kinetics fully account for the temporal difference between cholinergic and GABAergic transmission. Furthermore, GABA_A_ receptor blockade prolonged cholinergic transmission, identifying a new functional role for GABAergic inhibition of SACs. Thus, fast cholinergic transmission from SACs to DSGCs arises from at least two distinct mechanisms, yielding temporal properties consistent with conventional synapses despite its paracrine nature.

## Introduction

The direction-selective (DS) circuit in the mammalian retina represents a model system for linking a sensory computation to its underlying cellular and synaptic mechanisms. At the output of this circuit, DS ganglion cells (DSGCs) fire strongly to motion in a “preferred” direction and weakly to motion in the opposite, “null” direction (Barlow and Levick, 1965; Oyster, 1968). Critical to DSGC output is the convergence of three major synaptic inputs, provided by bipolar cells (BCs) and starburst amacrine cells (SACs). BCs, which receive direct input from photoreceptors, provide untuned (i.e., non-DS) excitatory glutamatergic input to DSGCs (Yonehara et al., 2013; Chen et al., 2014; Park et al., 2014; but see Matsumoto et al., 2019; Percival et al., 2019). BCs also provide excitatory glutamatergic input to SACs, which drives the release of both excitatory acetylcholine (ACh) and inhibitory GABA onto DSGCs (Brecha et al., 1988; Vaney and Young, 1988; O'Malley et al., 1992; Fried et al., 2002; Lee et al., 2010). Cholinergic input from SACs is untuned (Park et al., 2014; Sethuramanujam et al., 2016), acting in concert with glutamatergic BC input to provide non-DS excitation to DSGCs. GABAergic input from SACs to a DSGC, however, is strongly tuned to the DSGC's null direction (Fried et al., 2002; Lee et al., 2010; Wei et al., 2011), acting to counter excitatory input and, consequently, suppress DSGC spiking in response to stimulus motion in the null direction.

Each SAC neurite is depolarized preferentially by centrifugal motion [i.e., motion from the soma toward the distal tips of the neurite (Euler et al., 2002; Hausselt et al., 2007; Chen et al., 2016; Vlasits et al., 2016; Koren et al., 2017; Morrie and Feller, 2018; Poleg-Polsky et al., 2018), where synaptic release sites are located (Famiglietti, 1991; Ding et al., 2016)]. The non-directional nature of cholinergic input to DSGCs would predict that a DSGC receives synapses from SAC neurites oriented in all directions, with GABA transmitted only at synapses from null-directed neurites (Lee et al., 2010). Instead, a DSGC receives synapses predominantly from SAC neurites oriented in the DSGC's null direction (Briggman et al., 2011), generating the hypothesis that DSGCs receive direction-tuned GABAergic input from SACs via conventional synapses but untuned cholinergic input via paracrine (i.e., non-synaptic) transmission (Briggman et al., 2011; Sethuramanujam et al., 2016; Brombas et al., 2017; Hanson et al., 2019).

In paracrine transmission, released transmitters diffuse relatively long distances to their target receptors, which should prolong the time course of postsynaptic signals (Barbour et al., 1994; Digregorio et al., 2002; Nielsen et al., 2004; Szapiro and Barbour, 2007). Yet, in the retinal DS circuit, cholinergic transmission appears to be more transient than GABAergic transmission (Lee et al., 2010; Sethuramanujam et al., 2016; Hanson et al., 2019). This paradox is functionally relevant because the DS computation depends, in part, on the temporal mismatch between transient cholinergic excitation and prolonged GABAergic inhibition (Sethuramanujam et al., 2016; Hanson et al., 2019). Despite the computational significance of fast non-synaptic cholinergic transmission in this circuit, though, the mechanisms that enable it have yet to be fully elucidated.

Therefore, we developed an approach combining cell type-specific optogenetic stimulation, whole-cell electrophysiology, and linear systems analysis to examine mechanisms generating fast cholinergic transmission from SACs to DSGCs. While pharmacologically blocking endogenous light responses, we optogenetically evoked transmitter release from SACs using white-noise stimuli and recorded the resulting cholinergic or GABAergic postsynaptic currents (PSCs) in ON-OFF DSGCs. Under these conditions, cholinergic transmission exhibited faster kinetics than GABAergic transmission. Measurements of unitary PSCs were combined with a deconvolution-based analysis to reveal that transmitter-specific differences in postsynaptic receptor kinetics underlie the overall difference in transmission kinetics. Independently, GABA_A_ receptor blockade prolonged cholinergic transmission, identifying a role for presynaptic GABA_A_ receptors—likely activated by mutual inhibition between SACs—in modulating the kinetics of ACh release from SACs. Together, these results demonstrate the roles of specific receptors in shaping the temporal properties of cholinergic transmission and, additionally, suggest constraints on the cellular architecture of paracrine ACh transmission in retinal DS circuitry.

## Materials and Methods

### Animals

All animal procedures were approved by the Institutional Animal Care and Use Committee at Yale University and were in compliance with National Institutes of Health guidelines. Mice of both sexes were maintained on a C57BL/6 background and studied between postnatal days 28 and 90 (P28-P90). For all experiments, homozygous ChAT-IRES-Cre mice (B6;129S6-Chat^tm2(cre)Lowl^/J; The Jackson Laboratory #006410) were crossed with homozygous Ai32 mice (Madisen et al., 2012; B6.Cg-Gt(ROSA)26Sor^tm32(CAG−COP^4^^*^H134*R*/*EYFP*)*Hze*^/J; The Jackson Laboratory #024109) to produce offspring that were heterozygous for each transgene. In retinas of these mice, Cre expression is driven by endogenous ChAT regulatory elements and enables selective expression of a channelrhodopsin-2 (ChR2)/enhanced yellow fluorescent protein (EYFP) fusion protein in ON and OFF SACs.

### Electrophysiology

Mice were dark-adapted for ~1 h before euthanasia, immediately after which both eyes were enucleated and transferred to a dissection dish filled with Ames medium (A1420, Sigma-Aldrich) supplemented with 22.6 mM NaHCO_3_ (Sigma-Aldrich) and suffused with 95% oxygen/5% carbon dioxide at room temperature. Retinal dissections were performed under infrared illumination using stereomicroscope-mounted night vision goggles (B.E. Meyers). Following extraction of the retina from the eyecup, the vitreous humor was removed and a deep relaxing cut was made along the nasotemporal axis toward the optic disc. Retinas were mounted onto mixed cellulose ester filter membranes (HAWP01300, EMD Millipore) and maintained in the dissection dish at room temperature for up to 5 h until recording. Immediately prior to recording, mounted retinas were placed in a custom recording chamber and secured beneath a tissue harp. During experiments, the recording chamber was perfused with Ames medium flowing at 4–6 mL/min and maintained at 32–34°C.

Electrophysiological recordings were obtained using patch pipettes pulled from borosilicate glass capillaries (1B120F-4, World Precision Instruments). Pipette tip resistances were 4–6 MΩ for ganglion cell recordings and 5–8 MΩ for amacrine cell recordings. Pipettes were filled with internal solutions containing the following (in mM): 120 K-methanesulfonate, 10 HEPES, 0.1 EGTA, 5 NaCl, 4 ATP-Mg, 0.4 GTP-Na_2_, and 10 phosphocreatine-tris_2_, at pH 7.3 and 280 mOsm for current-clamp recordings; or 120 Cs-methanesulfonate, 5 TEA-Cl, 10 HEPES, 10 BAPTA, 3 NaCl, 2 QX-314-Cl, 4 ATP-Mg, 0.4 GTP-Na_2_, and 10 phosphocreatine-tris_2_, at pH 7.3 and 280 mOsm for voltage-clamp recordings. In a subset of recordings, the internal solution was supplemented with 0.05% (w/v) Lucifer yellow to fluorescently label cells for subsequent immunohistochemistry and visualization (Park et al., 2015, 2018). All compounds included in internal solutions were acquired from Sigma-Aldrich. During all recordings, membrane potential or current was amplified (MultiClamp 700B, Axon Instruments), digitized at 5 or 10 kHz (Digidata 1440A, Molecular Devices), and recorded (pClamp 10.0, Molecular Devices). During voltage-clamp recordings, excitatory or inhibitory currents were isolated by clamping at the reversal potential for chloride (E_Cl_; ~−67 mV) or cations (E_cations_; ~0 mV), respectively. Series resistance (10–25 MΩ) was compensated by 50%, and recordings were corrected for a −9-mV liquid junction potential.

To identify unlabeled DSGCs, loose-patch spike recordings were obtained from ganglion cells while visual stimuli were presented by a modified video projector (λ_peak_ = 395 nm) focused through a sub-stage condenser lens onto the retina (Borghuis et al., 2013, 2014). The mean luminance of visual stimuli was typically ~10^4^ photoisomerizations cone^−1^ s^−1^ (Borghuis et al., 2014). ON-OFF DSGCs and ON DSGCs were initially identified and distinguished by their categorically distinct spike responses to a ~5-s spot stimulus (400-μm diameter) of positive contrast: ON-OFF DSGCs fired transiently at stimulus onset and offset, whereas ON DSGCs responded with sustained firing that continually decayed over the course of stimulus presentation (Weng et al., 2005; Sun et al., 2006; Dhande et al., 2013). Most putative DSGCs were also presented with drifting grating stimuli to test direction selectivity (Park et al., 2014). During subsequent voltage-clamp recordings, DSGC identity was confirmed by the presence of both IPSCs and EPSCs during optogenetic stimulation of ChR2^+^ SACs (Sethuramanujam et al., 2016). Fluorescent dye-filled cells that exhibited these physiological properties always visibly co-stratified their dendrites with the EYFP^+^ neurites of SACs, as was further indicated by subsequent immunostaining against choline acetyltransferase (Park et al., 2015, 2018). To target ON SACs, EYFP^+^ somata in the ganglion cell layer were visualized using a custom-built two-photon laser-scanning microscope that was controlled by ScanImage (Vidrio Technologies) (Borghuis et al., 2013). Two-photon excitation was provided by a tunable Coherent Chameleon Ultra II laser (λ_peak_ = 910 nm).

During optogenetic experiments, ChR2^+^ SACs were photostimulated using an LED (λ_peak_ = 470 nm; M470L3, Thorlabs) projected through the aperture (400-μm diameter) of an iris diaphragm (CP20S, Thorlabs), driven by a T-Cube LED driver (LEDD1B, Thorlabs), and focused through a sub-stage condenser lens onto the retina. The maximum light intensity (Φ_max_) at the sample plane was 4.8 × 10^17^ quanta cm^−2^ s^−1^. Optogenetic stimuli were gamma-corrected to account for a nonlinear relationship between voltage input to the LED driver and light output of the LED, as measured at the sample plane. Conventional photoreceptor-mediated input was pharmacologically blocked via bath application of the following drug cocktail (in μM): 50 D-AP5 (Alomone), 50 DNQX (Alomone), 20 L-AP4 (Alomone), and 2 ACET (Tocris) (Park et al., 2015, 2018). For a subset of experiments, voltage-gated calcium channels were blocked by adding 200 μM CdCl_2_ (Sigma-Aldrich) to the cocktail described above, and Ames medium was replaced by a Ringer solution consisting of the following (in mM): 120 NaCl, 3.1 KCl, 1.15 CaCl_2_, 1.24 MgSO_4_, 6 glucose, and 22.6 NaHCO_3_. All compounds included in the Ringer solution were obtained from Sigma-Aldrich.

### Linear-Nonlinear Cascade Analysis

Linear-nonlinear (LN) cascade analysis was performed as previously described (Jarsky et al., 2011). Quasi-white-noise (WN) stimuli comprised 10 consecutive 10-s trials (100 s total), each consisting of 7.5 s of a unique sequence followed by 2.5 s of a repeated sequence. For each cell, responses to unique stimuli were used to generate an LN model while responses to repeated stimuli were used to test the predictive accuracy of the model. WN stimuli were initially generated by repeated draws from a Gaussian distribution and subsequently filtered to emphasize low, physiologically-relevant frequencies. Due to low-pass filtering associated with ChR2-mediated modulation of graded membrane potential (Tchumatchenko et al., 2013; Figure 2) and to the gradual reduction of postsynaptic current (PSC) reliability during continuous ChR2 stimulation (Figure 3F), WN stimuli were ideally low-pass filtered at 30 Hz (i.e., higher frequencies were removed). This low-pass filtering increased the signal-to-noise ratio of whole-cell recordings and thereby enabled construction of LN models from individual cells across all conditions; however, this filtering also generated ringing in linear filters. For analysis, stimuli were mean-subtracted and normalized by the maximum amplitude.

For each WN recording, trial-to-trial reliability (i.e., similarity) was measured using responses to the repeated stimulus sequence. Specifically, for the *i*^th^ of *n* trials, similarity (*s*_*i*_) was computed as the Pearson correlation coefficient (ρ) between that trial's repeat response (*r*_*i*_) and the mean of all other trials' repeat responses (${\bar{r}}_{i}$):

$$\begin{array}{l} {\bar{r}}_{i}=\frac{1}{n-1}\left[\left(\overset{n}{\underset{k=1}{\sum }}r_{k}\right)-r_{i}\right]\\ s_{i}=\rho_{r_{i},{\bar{r}}_{i}} \end{array}$$

These coefficients *s*_*i*_ were then averaged to compute an overall measure of reliability $\bar{s}$.

A linear filter *f*(*t*) is typically obtained by cross-correlation of a stimulus *s*(*t*) with a response *r*(*t*) followed by deconvolution with the stimulus autocorrelation, as shown here in the frequency (ω) domain:

$$\begin{array}{l} f\left(t\right)=F^{-1}\left[\frac{{\hat{s}}^{{\;}^{*}}\left(\omega \right)\hat{r}\left(\omega \right)}{{\hat{s}}^{{\;}^{*}}\left(\omega \right)\hat{s}\left(\omega \right)}\right]=F^{-1}\left[\frac{{\hat{s}}^{{\;}^{*}}\left(\omega \right)\hat{r}\left(\omega \right)}{S\left(\omega \right)}\right] \end{array}$$

where $F^{-1}$ is the inverse Fourier transform operator, ŝ(ω) is the Fourier transform of *s*(*t*), $\hat{r}\left(\omega \right)$ is the Fourier transform of *r*(*t*), ^*^ indicates complex conjugation, and *S*(ω) is the Fourier transform of the stimulus autocorrelation. However, because *S*(ω) contained very low power at high frequencies due to stimulus design, division by *S*(ω) amplified high frequency noise present in $\hat{r}\left(\omega \right)$, which heavily contaminated *f* (*t*). Therefore, we omitted this division step and instead computed the linear filter as $f\left(t\right)=F^{-1}\left[{\hat{s}}^{{\;}^{*}}\left(\omega \right)\hat{r}\left(\omega \right)\right]$. Linear filter width was measured as the full width at 25% of the maximum. The linear filter *f* (*t*) was then convolved with the stimulus *s* (*t*) to generate a linear prediction *r*_*L*_ (*t*) of the recorded response *r* (*t*). Equivalently:

$$\begin{array}{l} r_{L}\left(t\right)=F^{-1}\left[\hat{f}\left(\omega \right)\hat{s}\left(\omega \right)\right] \end{array}$$

where $\hat{f}\left(\omega \right)$ is the Fourier transform of *f* (*t*). Next, for each time point *t*_*i*_, *r* (*t*_*i*_) was plotted against *r*_*L*_ (*t*_*i*_) to directly compare the recorded and predicted responses. Plotted points were divided into 100 bins along the linear prediction (*x*-) axis, with each bin containing an equal number of points. Within each bin, points were averaged along both the recorded and predicted response dimensions. The resulting 100 points were then fit with a Gaussian cumulative distribution function *N* (*x*):

$$\begin{array}{l} N\left(x|\alpha ,\sigma ,\mu ,\delta \right)=\frac{\alpha }{\sigma \sqrt{2\pi }}\int_{-\infty }^{x}e^{\frac{-{\left(\tau -\mu \right)}^{2}}{2\sigma^{2}}}d\tau +\delta \end{array}$$

where α and σ scale *N* vertically and horizontally, respectively; and δ and μ offset *N* vertically and horizontally, respectively. *N* (*x*) was then used to convert the linear prediction *r*_*L*_ (*t*) into the final LN response prediction *r*_*LN*_ (*t*):

$$\begin{array}{l} r_{LN}\left(t\right)=N\left[r_{L}\left(t\right)\right] \end{array}$$

To quantify the rectification of the modeled response, a rectification index *i*_*rect*_ was computed using *N* (*x*) as follows:

$$\begin{array}{l} i_{rect}=\frac{\left|N\left[\max\left(r_{L\left[bin\right]}\right)\right]+N\left[\min\left(r_{L\left[bin\right]}\right)\right]-2N\left(0\right)\right|}{N\left[\max\left(r_{L\left[bin\right]}\right)\right]-N\left[\min\left(r_{L\left[bin\right]}\right)\right]} \end{array}$$

where *r*_*L*[*bin*]_ is the set of 100 values obtained after binning and averaging along the linear prediction axis, as described above.

The predictive accuracy of an LN model was measured by comparing the predicted and recorded responses to the repeated stimulus sequence. Specifically, the squared Pearson correlation coefficient (*r*^2^) was computed between (1) the predicted response to the repeated stimulus, generated by the LN model; and (2) the mean of all responses recorded during 10 trials of the repeated stimulus. For all conditions studied using LN analysis, these *r*^2^ values are reported in the corresponding figures.

### Event Analysis

Evoked monophasic IPSCs (emIPSCs) were recorded in DSGCs following brief (<10 ms) optogenetic stimulation of ChR2^+^ SACs. For each cell, stimulus intensity and duration were empirically determined such that roughly one-third of trials evoked no IPSC event. For each trial, the recorded trace was band-pass filtered and then thresholded to detect the rapid rising phase of an evoked IPSC. Thresholds were typically defined as 4 or 5 times the standard deviation of the pre-stimulus baseline of the filtered trace. Trials were discarded if no suprathreshold rising phase was detected (failure) or if multiple discrete rising phases were detected (multiphasic IPSC). Trials containing a single suprathreshold rising phase indicated a monophasic IPSC. Prior to analysis, emIPSCs were aligned to the first point at which the filtered trace exceeded the detection threshold. For each emIPSC, amplitude was measured as the peak of each event. Additionally, the time constant of decay (τ_decay_) was measured by fitting an exponential function to the decay phase of each emIPSC. Similar detection and analysis procedures were applied to spontaneous GABAergic IPSCs and cholinergic EPSCs. The averages of all unitary IPSCs and EPSCs were computed separately and fit with functions *f*(*t*) of the following forms:

$$\begin{array}{l} f_{EPSC}\left(t\right)=\left(a_{1}e^{-\frac{t}{\tau_{1}}}\right)\left(1-a_{2}e^{-\frac{t}{\tau_{2}}}\right)\\ f_{IPSC}\left(t\right)=\left(a_{1}e^{-\frac{t}{\tau_{1}}}+{a_{2}e}^{-\frac{t}{\tau_{2}}}\right)\left(1-a_{3}e^{-\frac{t}{\tau_{3}}}\right) \end{array}$$

where *a*_*i*_ are amplitude-scaling constants and τ_*i*_ are time constants. To facilitate comparison to the time courses of WN-derived linear filters, the full width at 25% of the maximum also was measured for f_EPSC_ and f_IPSC_.

Because spontaneous cholinergic EPSCs have lower amplitudes and signal-to-noise ratios than GABAergic emIPSCs (Figures 4A–C), there is more uncertainty associated with identification of their onsets and peaks. Specifically, because an sEPSC cannot be detected until it has risen above the noise, there is an expected bias toward late detection and, as a result, underestimation of the latency to peak. The function fit to the sEPSC average (Figure 4D) and used in the hybrid EPSC analysis (Figure 5) does not account for this source of uncertainty and/or bias in the peak time. The expected effects on measurement of hybrid EPSC filters would be systematic underestimation of both (1) the mean of the filter peak times and (2) the error associated with the mean estimate (Figure 5D1), with virtually no effect on filter width measurements (Figure 5D2).

### Wiener Deconvolution

Wiener deconvolution was used to estimate presynaptic dynamics from IPSCs recorded in ON-OFF DSGCs during optogenetic WN stimulation of ChR2^+^ SACs. This procedure assumes approximately linear summation of unitary events, which is supported by a weak correlation between amplitude and decay time constant in emIPSCs recorded in DSGCs (Kendall's τ coefficient = 0.025, *p* = 0.596; Figure 4C); i.e., emIPSC kinetics were approximately invariant across amplitudes (James et al., 2019).

In the context of this study, an optogenetic WN-evoked IPSC *i*(*t*) was modeled as the convolution of a presynaptic vesicle release record *r*(*t*) with an empirically-determined postsynaptic low-pass filter *p*(*t*) (see Figures 4, 5), plus noise *n*(*t*):

$$\begin{array}{l} i\left(t\right)=\left(r*p\right)\left(t\right)+n\left(t\right) \end{array}$$

In standard deconvolution, an estimate of *r*(*t*) is recovered by deconvolving *i*(*t*) with *p*(*t*). In the frequency (ω) domain, this can be equivalently expressed as

$$\begin{array}{l} ř\left(\omega \right)=\frac{\hat{i}\left(\omega \right)}{\hat{p}\left(\omega \right)}=\hat{i}\left(\omega \right)\frac{1}{\hat{p}\left(\omega \right)}=\hat{i}\left(\omega \right)\hat{q}\left(\omega \right) \end{array}$$

where ř(ω) is an estimate of the Fourier transform of *r*(*t*), î(ω) is the Fourier transform of *i*(*t*), $\hat{p}\left(\omega \right)$ is the Fourier transform of *p*(*t*), and $\hat{q}\left(\omega \right)$ is the Fourier transform of the inverse of *p*(*t*). However, inversion of a low-pass filter generates a high-pass filter, which amplifies high-frequency noise present in *i*(*t*). Wiener deconvolution reduces this noise amplification by weighting $\hat{q}\left(\omega \right)$ according to the signal-to-noise ratio (SNR) at each frequency ω:

$$\begin{array}{l} \hat{q}\left(\omega \right)=\hat{q}\left(\omega \right)\left[\frac{|\hat{p}\left(\omega \right){|}^{2}}{|\hat{p}\left(\omega \right){|}^{2}+\frac{N\left(\omega \right)}{S\left(\omega \right)}}\right]=\hat{q}\left(\omega \right)\left[\frac{|\hat{p}\left(\omega \right){|}^{2}}{|\hat{p}\left(\omega \right){|}^{2}+\frac{1}{SNR\left(\omega \right)}}\right] \end{array}$$

where $\hat{q}\left(\omega \right)$ is the Fourier transform of the SNR-weighted inverse filter and *N*(ω) and *S*(ω) are the power spectral densities of *n*(*t*) and *r*(*t*), respectively. î(ω) and $\hat{p}\left(\omega \right)$ were obtained by Fourier transformation of *i*(*t*) and *p*(*t*), respectively, which were acquired experimentally. To estimate *N*(ω), a 1-s period of the recording prior to stimulus onset was used to construct an amplitude histogram, which was then fit with a Gaussian distribution of variance $\sigma_{N}^{2}$. For Gaussian-distributed noise with variance $\sigma_{N}^{2}$, $N\left(\omega \right)=\sigma_{N}^{2}$ for all ω. *S*(ω) was estimated as the power spectral density of a Savitzky-Golay low-pass filtered version of *i*(*t*) (James et al., 2019). These terms jointly enabled calculation of ř(ω) via Wiener deconvolution:

$$\begin{array}{l} ř\left(\omega \right)=\hat{i}\left(\omega \right)\overset{\vee }{q}\left(\omega \right)=\frac{\hat{i}\left(\omega \right)}{\hat{p}\left(\omega \right)}\left[\frac{|\hat{p}\left(\omega \right){|}^{2}}{|\hat{p}\left(\omega \right){|}^{2}+\frac{\sigma_{N}^{2}}{S\left(\omega \right)}}\right] \end{array}$$

Subsequently, ř(ω) was utilized in two ways. For visualization of presynaptic dynamics (Figure 5A), inverse Fourier transformation of ř(ω) generated ř(*t*), which consisted of a series of δ function-like events of variable amplitude that presumably correspond to rapid bursts of vesicle release (James et al., 2019). For LN analysis of hybrid EPSCs (Figures 5B–D), ř(ω) was first multiplied by the Fourier transform of a function previously fit to the sEPSC average, and the result was subjected to inverse Fourier transformation to generate hybrid EPSCs.

### Statistics

Consistent with comparable studies and conventions in the field, each group included 4–10 cells from at least two mice of either sex. Unless otherwise stated, summary values are reported as mean ± SEM and statistical comparisons were performed using two-tailed Student's *t*-tests. Exact *p*-values are reported up to *p* < 0.001. Statistical significance levels are indicated in figures as follows: ^*^p < 0.05, ^**^p < 0.01, and ^***^p < 0.001.

## Results

### Channelrhodopsin-2 Enables Physiological, Reliable, and Dynamic Control of Synaptic Transmission From Starburst Amacrine Cells

**Figure 1:**
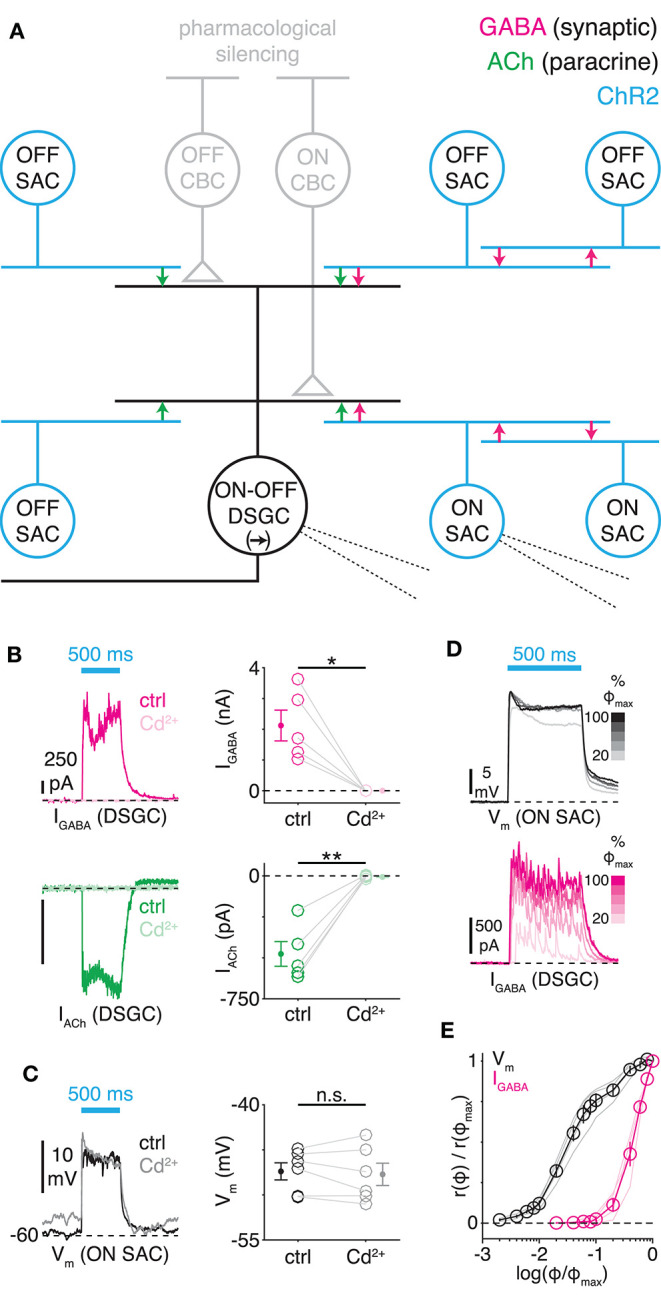
Optogenetic stimulation of starburst amacrine cells evokes postsynaptic currents in ON-OFF direction-selective ganglion cells. **(A)** An ON-OFF DSGC receives excitatory cholinergic and inhibitory GABAergic input from ON and OFF SACs. Neighboring SACs also provide GABAergic inhibition to each other. SACs express channelrhodopsin-2 (ChR2, blue), which is activated after blocking conventional glutamatergic transmission from photoreceptors and cone bipolar cells (CBCs; see Materials and Methods). **(B)** ChR2-evoked postsynaptic currents (PSCs) depend on voltage-gated calcium channels. *Left*, ChR2-evoked GABAergic IPSCs (I_GABA_) and cholinergic EPSCs (I_ACh_) recorded in separate ON-OFF DSGCs before (ctrl) and during bath application of cadmium (Cd^2+^, 200 μM). Stimulus intensity, 4.8 × 10^17^ quanta (Q) cm^−2^ s^−1^. *Right*, peak amplitude of ChR2-evoked PSCs before and during Cd^2+^ application (*n* = 5 cells). I_GABA_–ctrl vs. Cd^2+^: *p = 0.014, *t* = 4.2; I_ACh_–ctrl vs. Cd^2+^: **p = 0.003, *t* = 6.6 (paired *t*-tests). Error bars here and below indicate ± SEM across cells. **(C)** ChR2-mediated modulation of SAC V_m_. *Left*, ChR2-modulated V_m_ of ON SAC before and during bath application of Cd^2+^. *Right*, mean ChR2-evoked depolarization before and during Cd^2+^ application (*n* = 6 cells). **(D)** Dynamic range of ChR2-mediated responses. Within a range of stimulus intensity [20-100% Φ_max_ (4.8 × 10^17^ Q cm^−2^ s^−1^)], ON SAC V_m_ remains saturated while IPSCs increase. **(E)** Normalized input-output relations for ChR2-modulated ON SAC V_m_ (*n* = 4 cells) and ON-OFF DSGC IPSCs (*n* = 4 cells); light traces indicate individual cells. r(Φ) is the response to a stimulus of intensity Φ.

Combined with pharmacological blockade of native light responses, channelrhodopsin-2 (ChR2) photostimulation has been used extensively to study synaptic connectivity between identified retinal neurons (Lagali et al., 2008; Lee et al., 2016; Tien et al., 2016, 2017; Kim and Kerschensteiner, 2017; Park et al., 2018; Jia et al., 2020), especially in DS circuitry (Yonehara et al., 2011; Beier et al., 2013; Lee et al., 2014; Krishnaswamy et al., 2015; Park et al., 2015; Sethuramanujam et al., 2016; Hanson et al., 2019). Though ChR2-based neural circuit analysis is well-established in this context, we sought to expand the use of ChR2 as a tool for quantitative study of the temporal dynamics of synaptic transmission, particularly that from SACs to ON-OFF DSGCs (Figure 1A).

Toward this goal, we first tested whether ChR2-evoked synaptic transmission from SACs depends on Ca^2+^ influx through voltage-gated calcium channels (VGCCs) [i.e., the conventional mechanism (Lee et al., 2010)], rather than through ChR2 itself (Nagel et al., 2003; Krishnaswamy et al., 2015). We found that ChR2-evoked inhibitory postsynaptic currents (IPSCs) and excitatory PSCs (EPSCs) recorded in DSGCs were blocked completely by cadmium (Cd^2+^), a non-selective VGCC blocker: peak IPSC and EPSC amplitudes were reduced by 100.0 ± 0.1% (*p* < 0.001, *t* = −800.1) and 99.7 ± 0.8% (*p* < 0.001, *t* = 123.5), respectively (Figure 1B). By contrast, ChR2-dependent graded depolarization of ON SAC membrane potential (V_m_) was unaffected by Cd^2+^ (*p* = 0.51, *t* = 0.71; Figure 1C). Here and below, we recorded ON SACs because their somas reside in the ganglion cell layer and are therefore more accessible to patch-clamp recording than OFF SACs (Figure 1A). We conclude that ChR2-evoked synaptic transmission from SACs to DSGCs is driven solely by Ca^2+^ influx through endogenous VGCCs in SACs.

Next, we calibrated our ChR2-activating light stimulus by determining the relationship between ChR2-dependent depolarization of ON SACs and ChR2-evoked IPSCs recorded in DSGCs. Lower light intensities depolarized somatic ON SAC V_m_ monotonically without evoking synaptic transmission, whereas higher intensities saturated somatic V_m_ while evoking monotonic increases in IPSC amplitude (Figure 1D). These results suggest that somatic and synaptic V_m_ differ (Figure 1E), as might be expected in an electrotonically complex cell such as the SAC, in which release sites are located within the tips of thin, highly branched neurites (Miller and Bloomfield, 1983; Ding et al., 2016). The discrepancy between somatic and synaptic V_m_ precluded a direct mapping of SAC V_m_ onto IPSC amplitude, and consequently, our experiments utilized stimulation intensities within the dynamic range of ChR2-evoked IPSCs.

**Figure 2:**
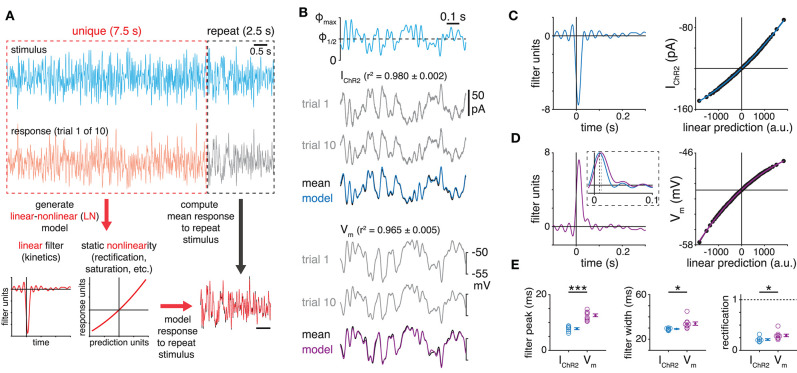
Reliability and kinetics of ChR2-mediated white-noise stimulation of starburst amacrine cells. **(A)** Construction of linear-nonlinear model from responses to optogenetic white-noise stimulation. **(B)** *Top*, optogenetic white-noise stimulus sequence. Maximum light intensity (Φ_max_), 3.2 × 10^17^ Q cm^−2^ s^−1^. *Middle*, ChR2 currents (I_ChR2_) recorded in an ON SAC. Gray traces show responses to the repeated stimulus (trials 1 and 10). The average of all 10 responses (black) is overlaid with the output of the LN model (blue) generated from responses to unique stimuli. *Bottom*, same as *middle* for ChR2-modulated membrane potential (V_m_) in a different ON SAC. **(C)** Linear filter (*left*) and static nonlinearity (*right*) obtained from recording I_ChR2_ in ON SAC shown in **(B)**. For the static nonlinearity, the horizontal line indicates the response corresponding to a linear prediction of 0 (in arbitrary units, a.u.). Solid curves are fits to measured nonlinearities (black points). **(D)** Same as **(C)** for V_m_ in ON SAC shown in **(B)**. Inset shows linear filters for I_ChR2_ (inverted) and V_m_ superimposed on an expanded timescale. Dashed lines indicate filter peak times. **(E)** Measurements of LN model components obtained from recordings of I_ChR2_ (*n* = 7 cells) or V_m_ (*n* = 9 cells) in ON SACs. *p < 0.05; ***p < 0.001.

To quantify temporal properties of SAC transmission, we adopted a linear-nonlinear (LN) cascade analysis-based approach (Chichilnisky, 2001) used previously to study visual adaptation at various stages of retinal circuitry (Kim and Rieke, 2001; Baccus and Meister, 2002; Zaghloul et al., 2003; Beaudoin et al., 2007; Jarsky et al., 2011). We first presented a stochastic, white-noise (WN) stimulus, filtered at 30 Hz (see Materials and Methods), to evoke depolarization of ChR2^+^ SACs and PSCs in DSGCs. From each response, we constructed an LN model consisting of a linear filter, describing the kinetics of the modeled response; and a static nonlinearity, capturing time-invariant response properties such as rectification and saturation (Figure 2A). The output of the LN model is generated by first convolving the linear filter with the stimulus and then passing the result through the static nonlinearity, which serves as a lookup table. By capturing nonlinear features in a separate stage, LN model-based analysis enables isolation of linear filtering properties. Below, we measure the width of the linear filter to quantify the kinetics of the modeled response (e.g., EPSCs), with a “narrower” filter indicating more transient kinetics (see Materials and Methods). Finally, in addition to their application in LN analysis, WN stimuli also feature complex temporal structure that better resembles naturalistic stimuli than do conventional pulsatile stimuli.

We first directly examined the ability of ChR2^+^ SACs to encode optogenetic WN stimuli. The fidelity and temporal bandwidth of ChR2-mediated depolarization in non-spiking neurons like SACs has been studied rarely (Tchumatchenko et al., 2013) and never in the context of an intact neural circuit. Optogenetic WN stimulation reliably and rapidly modulated ChR2-mediated current (I_ChR2_) and V_m_ recorded in ChR2^+^ SACs, eliciting remarkably stereotyped responses across repeated trials (I_ChR2_: reliability [$\overset{\bar{\;}}{s}$] = 0.985 ± 0.002; V_m_: $\overset{\bar{\;}}{s}$ = 0.943 ± 0.013; Figure 2B). Resulting LN models accurately predicted responses to a validation stimulus that was not used for model construction (Figure 2B). Linear filters revealed I_ChR2_ to be a modestly filtered representation of the stimulus, while V_m_ was a more strongly filtered representation: I_ChR2_ filters peaked earlier than V_m_ filters (*p* < 0.001, *t* = −7.7) and were also narrower (*p* = 0.011, *t* = −3.2; Figures 2C–E). The I_ChR2_ filter reflects the ChR2 conductance itself, whereas the wider V_m_ filter reflects the additional influence of the membrane time constant. For both signals, static nonlinearities exhibited mild rectification, which was slightly more pronounced in V_m_ than in I_ChR2_ (*p* = 0.016, *t* = 2.7; Figures 2D,E). This apparent rectification likely reflects partial saturation of the ChR2-mediated conductance at the soma, whereas the V_m_ at synapses likely exhibits less saturation within this stimulus range (Figures 1D,E). In summary, though, these results demonstrate that ChR2^+^ SACs reliably encode dynamic light stimuli.

### Transmitter-Specific Temporal Properties of SAC→DSGC Transmission

**Figure 3:**
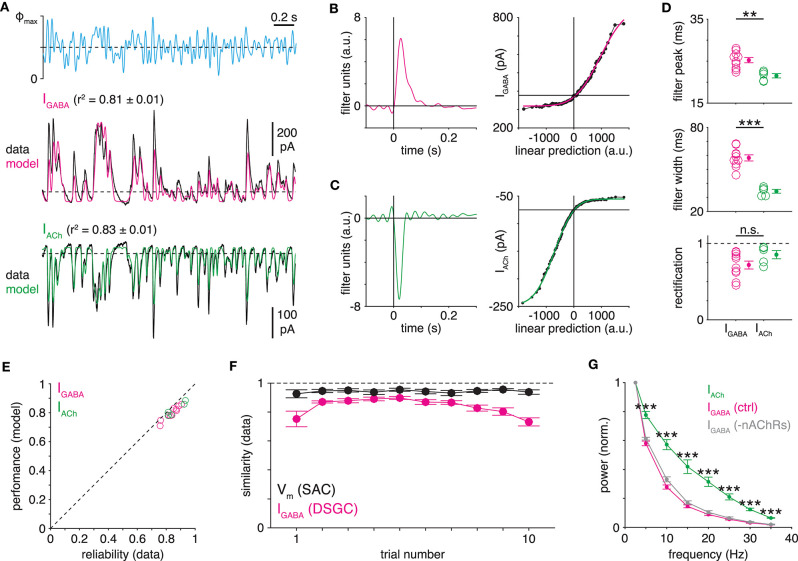
Transmitter-specific kinetics of SAC→DSGC transmission. **(A)** LN models obtained from IPSCs (I_GABA_) and EPSCs (I_ACh_) recorded in ON-OFF DSGCs during optogenetic white-noise stimulation of SACs. Black traces indicate averaged response to 10 stimulus repeats (Φ_max_ = 4.8 × 10^17^ Q cm^−2^ s^−1^). Colored traces show LN model output. Dashed horizontal lines indicate the response at a linear prediction of 0 [see **(B)**,**(C)**]. **(B,C)** Linear filters (*left*) and static nonlinearities (*right*) obtained from IPSCs **(B)** and EPSCs **(C)** shown in **(A)**. Horizontal line overlaid on each static nonlinearity indicates the response at a linear prediction of 0; this value is used to calculate rectification (see Materials and Methods). **(D)** Measurements of LN model components obtained from IPSCs (*n* = 10 cells) and EPSCs (*n* = 5 cells) recorded in ON-OFF DSGCs. **(E)** Relation between LN model performance and data reliability [average similarity; see **(F)**, Materials and Methods] for all PSC recordings. Dashed line indicates unity. **(F)** Similarity of ChR2-evoked responses to repeated stimuli on a trial-by-trial basis for SAC V_m_ (*n* = 9 cells; black) and ON-OFF DSGC IPSC (*n* = 10 cells; I_GABA_, magenta). Similarity is the Pearson correlation coefficient between the response during the *n*th trial and the mean of responses during all other trials. **(G)** Frequency analysis of IPSCs and EPSCs recorded in ON-OFF DSGCs. IPSCs were recorded in the absence (ctrl, magenta) or presence (gray) of hexamethonium (50 μM), a nicotinic ACh receptor (nAChR) antagonist. Prior to averaging across cells, each power spectrum was normalized to its power at 2.5 Hz. Power spectra are derived from the entire 100-s WN PSC recording. **p < 0.05; ***p < 0.001.

We combined WN light stimulation of ChR2^+^ SACs with LN analysis to compare computational properties of GABAergic and cholinergic transmission from SACs to DSGCs. The two transmitter systems exhibited significant differences in temporal filtering: compared to linear filters obtained from GABAergic IPSCs, filters from cholinergic EPSCs peaked earlier (*p* = 0.002, *t* = −3.9) and were narrower (*p* < 0.001, *t* = −7.3; Figures 3A–D). EPSCs and IPSCs exhibited similar rectification (*p* = 0.126, *t* = 1.63; Figures 3A–D). Similar to LN models of spike responses of ChR2^+^ GCs to optogenetic WN stimuli (Ferrari et al., 2020), our LN models exhibited high predictive accuracy that was limited primarily by the reliability of PSCs across trials (Figure 3E). Notably, PSC reliability decreased gradually during continuous stimulation, despite the stability of presynaptic V_m_ (Figure 3F). Therefore, we restricted our recordings to a stimulus period (100 s) during which PSCs were relatively stable.

We also performed an additional analysis to evaluate, independently of LN modeling, the possibility of transmitter-specific temporal filtering. Indeed, normalized power spectra computed directly from each raw PSC recording confirmed that, compared to GABAergic transmission, cholinergic transmission better passes high-frequency stimulus components to DSGCs (*p* < 0.001; Figures 3A,G). Because a subset of GABAergic and glycinergic ACs express nicotinic ACh receptors (nAChRs) (Dmitrieva et al., 2001, 2003, 2007), we also tested whether the relative prolongation of IPSCs could be explained by a polysynaptic circuit (SAC[ACh]→non-SAC AC[GABA/glycine]→DSGC) that provides delayed inhibition to DSGCs. IPSCs recorded in the presence of the nAChR antagonist hexamethonium, however, yielded power spectra similar to controls (Figure 3G), suggesting that the observed temporal difference results instead from intrinsic synaptic mechanisms.

### Postsynaptic Basis for Transmitter-Specific Temporal Filtering

**Figure 4:**
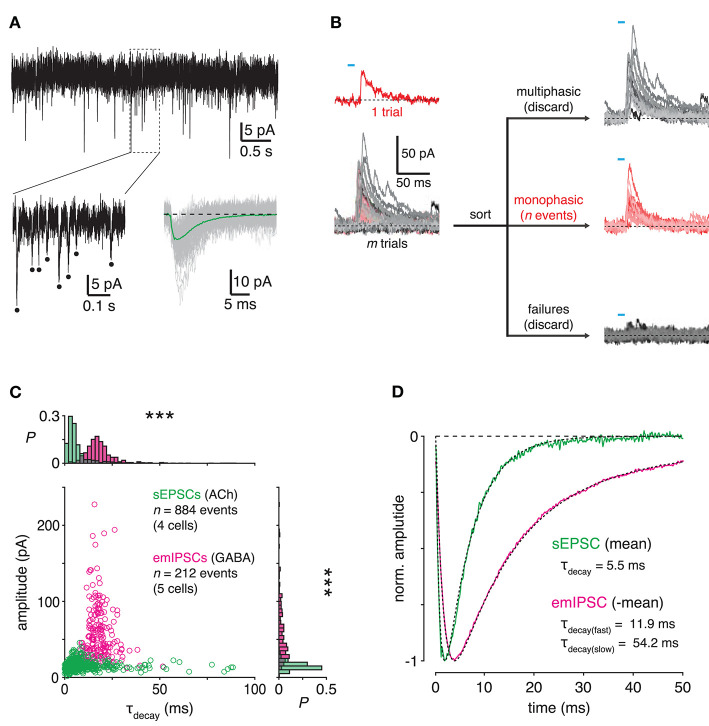
Transmitter-specific postsynaptic filtering in SAC→DSGC transmission. **(A)** Isolation and measurement of spontaneous cholinergic EPSCs in ON-OFF DSGCs. *Top*, spontaneous EPSCs (sEPSCs) recorded from an ON-OFF DSGC during blockade of AMPA (50 μM DNQX) and NMDA (50 μM D-AP5) receptors. *Bottom left*, expanded view of boxed region. Dots indicate individual spontaneous EPSCs. *Bottom right*, alignment and averaging of cholinergic sEPSCs. One hundred individual sEPSCs (light gray) superimposed with the mean (green) of all sEPSCs. Dotted line indicates baseline holding current. **(B)** Schematic diagram illustrating experimental generation and recording of evoked monophasic IPSCs (emIPSCs) in an ON-OFF DSGC. Brief (<10 ms) optogenetic stimulation of presynaptic SACs evokes a small, monophasic IPSC (red). Following *m* trials, *n* monophasic events are distinguished from multiphasic events and failures. **(C)** Comparison of all sIPSCs and emIPSCs recorded in DSGCs. Amplitude is plotted against decay time constant (τ_decay_) for all sIPSCs (*n* = 884 events in 4 ON-OFF DSGCs) and emIPSCs (*n* = 212 events from 4 ON-OFF DSGCs and 1 ON DSGC). Marginal probability distributions of amplitude and τ_decay_ are shown at right and above, respectively. PSC amplitude—sEPSC vs. emIPSC: ***p < 0.001, *D* = 0.82 (Kolmogorov-Smirnov test). **(D)** Averaged time courses of sIPSCs and emIPSCs. The average of all sIPSCs (green) is fit (black, dashed; see Materials and Methods) with a single exponential decay term. The average of all emIPSCs (inverted, magenta) is fit with two exponential decay terms. Traces are normalized to their respective maxima.

The time course of evoked PSCs (Figure 3) reflects the combined time courses of a sequence of at least three major pre- and postsynaptic processes: ChR2-mediated depolarization (Figure 2), presynaptic Ca^2+^ dynamics, and postsynaptic receptor kinetics. Therefore, we next sought to parse the relative contributions of pre- and postsynaptic mechanisms to the observed temporal difference between cholinergic and GABAergic transmission from SACs to DSGCs. To do so, we first assessed postsynaptic receptor kinetics by examining the waveforms of unitary EPSCs and IPSCs recorded from DSGCs. Because SACs provide the only cholinergic input to DSGCs, we estimated postsynaptic filtering in cholinergic SAC→DSGC transmission simply by measuring spontaneous EPSCs (sEPSCs) recorded in DSGCs while blocking ionotropic glutamate receptors (Figure 4A). Inhibitory input to DSGCs, however, arises from several AC types in addition to SACs (Park et al., 2015; Pei et al., 2015; Bleckert et al., 2018); thus, not all sIPSCs recorded in DSGCs originate from SACs. To resolve this ambiguity, we designed an experiment to evoke unitary IPSCs from SACs only. We briefly (<10 ms) stimulated ChR2^+^ SACs to evoke small, sIPSC-like events in DSGCs (4 ON-OFF DSGCs and 1 ON DSGC); we refer to these events as evoked monophasic IPSCs (emIPSCs; Figure 4B). Following automated detection, sorting, and alignment (see Materials and Methods), we measured individual and averaged sEPSCs and emIPSCs. Compared to cholinergic sEPSCs (*n* = 884 events from 4 cells), individual GABAergic emIPSCs (*n* = 212 events from 5 cells) exhibited waveforms with much slower decay kinetics (*p* < 0.001, *D* = 0.77, Kolmogorov-Smirnov test; Figure 4C). On average, cholinergic sEPSCs decayed rapidly (fit to mean sEPSC: τ_decay_ = 5.5 ms; width = 10.2 ms; Figure 4D). By contrast, the emIPSC average exhibited a prolonged “tail” during the decay phase—obscured by noise in individual emIPSCs—that was captured by a second, slow exponential decay term (τ_decay(fast)_ = 11.9 ms; τ_decay(slow)_ = 54.2 ms; width = 28.5 ms; Figure 4D). Spontaneous IPSCs recorded in the same DSGCs, on average, also exhibited a prolonged tail (τ_decay(fast)_ = 8.6 ms; τ_decay(slow)_ = 30.8 ms; width = 23.4 ms) but exhibited slightly faster decay kinetics than emIPSCs (*p* < 0.001, *D* = 0.245, Kolmogorov-Smirnov test; Supplementary Figure 1), likely reflecting a proportion of synapses from non-SAC ACs. Overall, these results suggest that postsynaptic mechanisms prolong GABAergic, relative to cholinergic, transmission from SACs to DSGCs.

**Figure 5:**
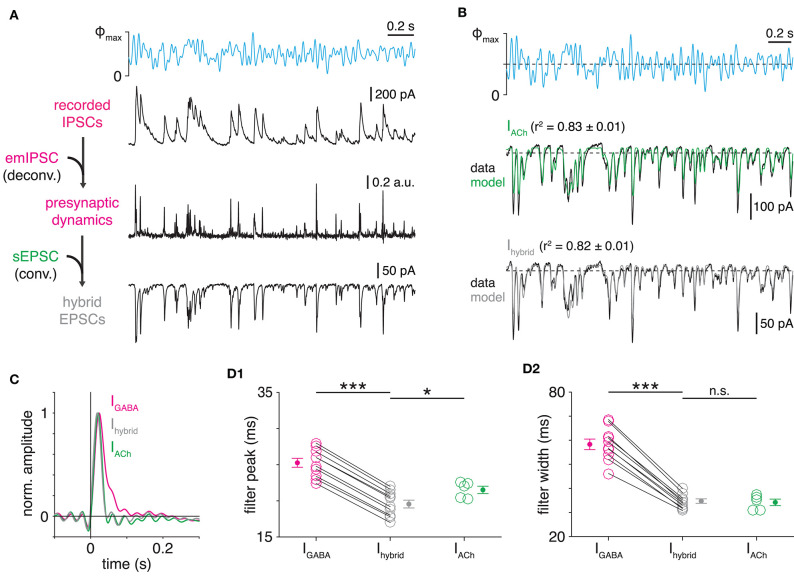
Differential postsynaptic kinetics explain transmitter-specific filtering in SAC→DSGC transmission. **(A)** Protocol for generation of hybrid PSCs. Wiener deconvolution of ON-OFF DSGC IPSCs removes the contribution of GABAergic postsynaptic kinetics (i.e., emIPSC time course) to estimate presynaptic release dynamics. The result is convolved with an estimate of cholinergic postsynaptic kinetics (i.e., sEPSC time course) to generate hybrid EPSCs. **(B)** Comparison of recorded and hybrid EPSC responses to optogenetic WN stimulation of SACs. *Top*, optogenetic WN stimulus. Maximum light intensity (Φ_max_), 4.8 × 10^17^ Q cm^−2^ s^−1^. Mean responses (black) and LN models (colored) for cholinergic EPSCs recorded in an ON-OFF DSGC (I_ACh_, *middle*) and hybrid EPSCs (I_hybrid_, *bottom*) combining i) presynaptic dynamics of GABAergic transmission in a different ON-OFF DSGC with ii) postsynaptic kinetics of cholinergic transmission. **(C)** Comparison of linear filters from recorded PSCs and hybrid EPSCs. Linear filters obtained from GABAergic IPSCs (magenta) and cholinergic EPSCs (green) recorded in separate ON-OFF DSGCs, overlaid with linear filter from hybrid EPSCs (gray) shown in **(B)**. For direct comparison, hybrid EPSC filter shown was generated using the IPSC recording whose filter is overlaid. **(D)** Peak times **(D1)** and widths **(D2)** of linear filters obtained from IPSCs (*n* = 10 cells) and EPSCs (*n* = 5 cells) recorded in ON-OFF DSGCs and hybrid EPSCs (*n* = 10 cells). Linear filter peak time—I_GABA_ vs. I_hybrid_: ***p < 0.001, *t* = 66.4; linear filter width—I_GABA_ vs. I_hybrid_: ***p < 0.001, *t* = 18.0 (paired *t*-tests). Recorded PSCs analyzed are from Figure 3. *p < 0.05.

To infer how much the observed differences in postsynaptic filtering (Figure 4) contribute to overall differences in transmitter-specific filtering observed in WN-evoked responses (Figure 3), we developed a deconvolution-based analysis. In this approach, we first used Wiener deconvolution to estimate instantaneous presynaptic release rates from GABAergic IPSCs recorded in ON-OFF DSGCs during WN stimulation of presynaptic SACs (Figure 5A; see Materials and Methods; James et al., 2019). We then generated “hybrid” EPSCs by convolving the estimated release rate at GABAergic SAC→DSGC synapses with the average cholinergic sEPSC (Figure 4D). These hybrid EPSCs were then subjected to LN analysis to extract temporal filters. If postsynaptic filtering (i.e., sEPSC waveform) accounts fully for transmitter-specific filtering at SAC synapses, then the linear filters of hybrid EPSCs should be indistinguishable from those of recorded EPSCs. Indeed, LN analysis revealed nearly identical filter widths for hybrid EPSCs compared to those for recorded EPSCs (*p* = 0.94, *t* = 0.074; Figures 5B–D). The peak times of filters for hybrid EPSCs were slightly shorter (by <2 ms) than those for recorded EPSCs, which was marginally significant (*p* = 0.047, *t* = −2.19; Figure 5D). While this difference in filter peaks could reflect a small but genuine delay in cholinergic transmission, it instead could be explained by the technical challenge of accurately determining the onset times of small sEPSCs (see Materials and Methods). Overall, though, these results suggest that differences in postsynaptic dynamics alone suffice to explain transmitter-specific filtering in SAC→DSGC transmission (Figure 5C).

### GABA_A_ Receptors Modulate Cholinergic Transmission Kinetics

**Figure 6:**
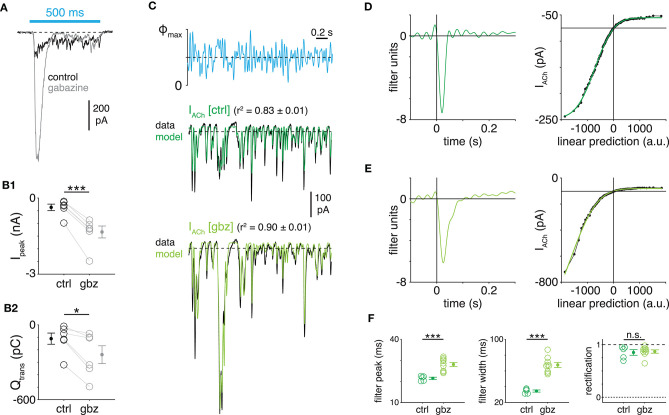
GABA_A_ receptors accelerate cholinergic SAC→DSGC transmission. **(A)** ChR2-evoked cholinergic EPSCs in an ON-OFF DSGC before (control, black) and during (gray) bath application of gabazine (25 μM). **(B)** Peak amplitude (I_peak_) **(B1)** band charge transfer (Q_trans_) **(B2)** of ChR2-evoked EPSCs recorded in ON-OFF DSGCs before (ctrl) and during gabazine application (gbz; *n* = 6 cells). **(C)** Linear-nonlinear models of ChR2-evoked cholinergic EPSCs (I_ACh_) recorded in the absence or presence of gabazine. **(D)** Linear filter (*left*) and static nonlinearity (*right*) obtained from a control recording of ChR2-evoked cholinergic EPSCs in an ON-OFF DSGC. **(E)** Same format as **(D)** in a different ON-OFF DSGC during application of gabazine. **(F)** Measurements of LN model components obtained from EPSCs recorded in the absence (ctrl; *n* = 5 cells) or presence (gbz; *n* = 9 cells) of gabazine. *p < 0.05; ***p < 0.001.

Visually-evoked Ca^2+^ influx into SAC neurites appears to be modulated by activation of GABA_A_ receptors (GABA_A_Rs) on these neurites, indicating a role for GABAergic inhibition in modulating the computation of direction selectivity (Lee and Zhou, 2006; Chen et al., 2016; Ding et al., 2016; Poleg-Polsky et al., 2018). Therefore, we examined how GABAergic inhibition might modulate temporal filtering of cholinergic transmission from SACs to ON-OFF DSGCs. Bath application of the GABA_A_R antagonist gabazine increased both the amplitude (*p* < 0.001, *t* = 8.8) and charge transfer (*p* = 0.016, *t* = 3.6) of ChR2-evoked cholinergic EPSCs (Figures 6A,B). Further, LN analysis of EPSCs evoked by WN stimulation revealed that GABA_A_R blockade slows the kinetics of cholinergic transmission: linear filters in the presence of gabazine were delayed (*p* < 0.001, *t* = 4.6) and wider (*p* < 0.001, *t* = 7.3) compared to filters measured under control conditions (Figures 6C–F). By contrast, GABA_A_R blockade did not visibly alter filtering properties of ChR2-evoked depolarization in ON SACs (Supplementary Figure 2), though GABA_A_R-dependent changes in synaptic V_m_ may simply be difficult to detect using somatic V_m_ recordings (Figures 1D,E). Rectification of cholinergic EPSCs was also insensitive to gabazine (*p* = 0.78, *t* = 0.29; Figures 6C–F). These results provide direct evidence that the kinetics of cholinergic transmission to DSGCs are regulated by GABA_A_Rs on presynaptic SACs.

## Discussion

### An Integrated Optogenetic and Computational Approach to Studying Retinal Synapses

We combined cell type-specific optogenetic stimulation, whole-cell electrophysiology, and linear systems analysis in a technical framework that permitted a quantitative study of synaptic transmission from interneurons in the mouse retina. Provided genetic access to a homogenous neuronal population, optogenetic stimulation confers multiple advantages over conventional paired recording. For example, all genetically-identified neurons presynaptic to a single postsynaptic neuron can be stimulated simultaneously, dramatically increasing the signal-to-noise ratio of recorded PSCs. Further, substituting ChR2 for a patch pipette and stimulating electrode circumvents dialysis of presynaptic neurons and, consequently, washout of any soluble components required for normal synaptic transmission. This approach also presents specific limitations, including the requirements of cell type-specific genetic access and pharmacological blockade of endogenous light responses. Notably, ChR2-evoked PSCs gradually (>100 s) decay during continuous WN stimulation (Figure 3F), limiting the duration over which PSCs can be evoked. PSC decay does not result from ChR2 desensitization (Figure 3F) and instead could be due to synaptic acidification caused by ChR2-mediated proton influx (Nagel et al., 2003; Lin et al., 2009) or to illumination-induced tissue heating, which can produce temperature-dependent physiological changes independent of an optogenetic actuator (Yizhar et al., 2011; Owen et al., 2019).

Using ChR2, we activated SACs with a quasi-WN light stimulus that spanned a physiologically- and ethologically-relevant range of temporal frequency (0–30 Hz) (Dong and Atick, 1995; Simoncelli and Olshausen, 2001; Wang et al., 2011). Linear systems analysis of PSCs evoked by optogenetic WN stimulation of SACs enabled quantitative comparison of temporal filtering implemented by cholinergic and GABAergic transmission to DSGCs. Application of LN cascade analysis isolated temporal filtering characteristics from static nonlinear features, such as rectification and saturation (Figure 3). We also developed a deconvolution-based analysis that generated an estimate of presynaptic release rate and, subsequently, hybrid PSCs; this analysis revealed the relative contributions of pre- and postsynaptic mechanisms to transmitter-specific synaptic filtering (Figure 5). Finally, though we compared two transmitter systems that convey signals from one presynaptic cell type to a second postsynaptic cell type, the technical and analytical framework described here can be adapted to a wide range of applications. This approach can be used, for example, to study the computational heterogeneity of synaptic outputs that diverge from one presynaptic cell type to multiple postsynaptic types; inversely, it can be applied to compare synaptic inputs that converge from multiple presynaptic cell types onto a single postsynaptic cell type.

### Mechanisms for Fast Cholinergic Transmission in the Retinal DS Circuit

**Figure 7:**
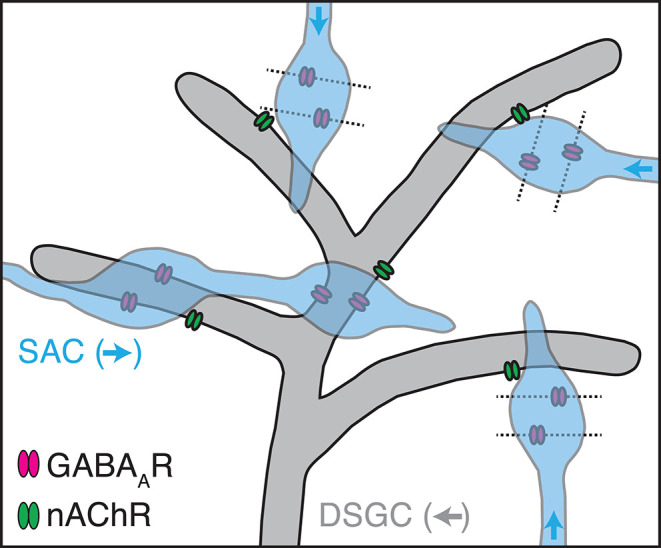
A cellular architecture consistent with fast, non-synaptic, and non-DS cholinergic transmission from SACs to DSGCs. Dendrites of a DSGC preferring leftward motion (gray) express GABA_A_ receptors (GABA_A_Rs, magenta) at conventional synapses from a SAC neurite preferring rightward motion (blue). Nearby, SAC neurites oriented in other directions provide GABAergic synapses onto dendrites of other DSGCs (dashed outlines). On the central DSGC, nicotinic ACh receptors (nAChRs, green) localize near any SAC varicosity positioned sufficiently close, regardless of its direction preference (blue arrows).

A central conclusion of this study is that cholinergic transmission from SACs to DSGCs exhibits a time course comparable to that of conventional synaptic transmission (Figure 3) despite the fact that the underlying synaptic architecture suggests it to be paracrine (Briggman et al., 2011). Indeed, SACs release ACh sufficiently close to postsynaptic nAChR clusters on DSGCs to generate detectable sEPSCs (Figure 4; Sethuramanujam et al., 2020). The discrepancy between untuned cholinergic EPSCs and the tuned orientation distribution of presynaptic SAC neurites in a DSGC argues against conventional cholinergic synapses; yet, the naturally compact arrangement of SAC output varicosities and DSGC dendrites apparently enables rapid, short-range transmission (Sethuramanujam et al., 2020). This model (Figure 7) also implies that nAChRs on a DSGC dendrite cluster near a SAC varicosity at a density sufficient to transduce sEPSCs. While such a quasi-synaptic architecture would be the principal basis for fast cholinergic transmission in DSGCs, our deconvolution-based analysis suggests that, given this architecture, postsynaptic receptor kinetics alone accelerate cholinergic transmission relative to GABAergic transmission (Figure 5D). By extension, cholinergic and GABAergic SAC→DSGC presynapses appear to be equivalent in their temporal filtering properties.

### A Role for GABA_A_ Receptors in Regulating Cholinergic Transmission Kinetics

We identified a novel function for GABAergic synapses onto SACs: modulating the kinetics, as well as the amplitude, of cholinergic transmission from SACs to DSGCs (Figure 6). Consistent with this result, GABAergic synapses from wide-field ACs modulate the amplitude of cholinergic transmission from OFF SACs to ON-OFF DSGCs in a stimulus-dependent manner (Huang et al., 2019). With GABA_A_ receptor signaling intact, differences in postsynaptic receptor kinetics fully explain differences in temporal filtering between cholinergic and GABAergic transmission (Figure 5D). Thus, presynaptic GABA_A_ receptors provide for independent regulation of cholinergic transmission dynamics. Although GABA_A_ receptors were blocked broadly in our experiments, it seems likely that SAC→SAC inhibition (Zheng et al., 2004; Lee and Zhou, 2006; Ding et al., 2016) mediates the observed effect on cholinergic transmission because (1) SACs were the only SAC-targeting GABAergic neurons stimulated directly using ChR2; (2) spontaneous IPSCs in SACs, which could originate from other AC types, are infrequent (>0.3 Hz) when glutamate receptors are blocked (Chen et al., 2016); and (3) SACs provide ~93% of GABAergic synapses onto other SACs (Ding et al., 2016). Alternatively, given that some GABAergic ACs express nAChRs in rabbit retina (Dmitrieva et al., 2001, 2007), it is also possible that ChR2-evoked ACh release from SACs drives a subset of these cholinoceptive ACs to release GABA back onto SACs in mice.

Regardless of which AC types provide the relevant GABAergic input, multiple mechanisms downstream from GABA_A_Rs on SACs could act to prolong cholinergic transmission to DSGCs during GABA_A_R blockade. For example, given that GABA_A_R blockade dramatically increases the amplitude of ChR2-evoked cholinergic EPSCs in our preparation (Figures 6A,B), it is likely that ACh release from SACs is increased under these conditions. Sufficiently amplified ACh release could saturate acetylcholinesterase, an extracellular hydrolase that breaks down released ACh, thereby prolonging the decay of extracellular ACh concentration. Additionally, increased ACh release could promote diffusion of ACh to distant nAChRs, with diffusion acting as a low-pass filter (DeVries et al., 2006). Enhanced low-pass filtering in cholinergic EPSCs also might be explained by an increase in vesicle release probability at SAC presynapses (Zucker and Regehr, 2002; Abbott and Regehr, 2004; Korber and Kuner, 2016). It is worth noting, finally, that bath-applied gabazine also blocks GABA_A_Rs on DSGC; in principle, this could increase the input resistance of a recorded DSGC enough to alter measurements of cholinergic EPSC kinetics, even without changes in ACh release from presynaptic SACs. This postsynaptic mechanism, however, is likely negligible given that blockade of nAChRs on DSGCs did not affect IPSC kinetics (Figure 3G).

### Consequences of Fast Cholinergic Transmission for Retinal DS Circuit Function

While the dominant mechanism for DS tuning in DSGCs is the null direction-tuned amplitude of GABAergic IPSCs generated by SACs (Fried et al., 2002; Wei et al., 2011), an additional mechanism is generated by a spatial offset in excitatory and inhibitory input fields (Hanson et al., 2019). For example, in a reduced preparation, DS tuning to optogenetic SAC stimulation persisted under conditions in which SAC GABA release was rendered non-DS by deletion of GABA_A_ receptors on SACs (Hanson et al., 2019). In this case, the GABAergic input field was spatially offset toward the DSGC's null side by ~25 μm relative to the cholinergic input field. Consequently, during null-direction motion, a stimulus first drives GABA release before subsequently driving ACh release. For inhibition to fully null the excitatory drive of ACh, though, GABAergic transmission must be sufficiently prolonged to interact with excitation evoked by stimulation of the preferred-side edge of the cholinergic input field (i.e., after the stimulus has exited the offset GABAergic input field).

Our reported difference in cholinergic and GABAergic synaptic filtering (~20 ms difference in filter width; Figure 3D) is consistent with computational models suggesting that temporal offsets as short as 10 ms can disrupt DSGC output (Jain et al., 2020). For example, given a 25-μm spatial offset between cholinergic and GABAergic input fields of a DSGC (Hanson et al., 2019), prolongation of GABAergic transmission by 20 ms would enable inhibition to fully outlast excitation at stimulus velocities above 1250 μm/s during null-direction motion (~42°/s, assuming 30 μm of retinal arc per 1° of visual angle; Ding et al., 2016). Furthermore, our estimated differences between linear filter widths likely represent lower bounds imposed by technical constraints related to continuous optogenetic stimulation (see Materials and Methods). Such interaction of temporal and spatial offsets between inputs to DSGCs could underlie a classical observation in rabbit retina: above some stimulus velocity threshold, null-direction motion evokes no spiking in DSGCs; at stimulus velocities below this threshold, however, weak null-direction spike responses emerge (Barlow and Levick, 1965).

## Data Availability Statement

The raw data supporting the conclusions of this article will be made available by the authors, without undue reservation.

## Ethics Statement

The animal study was reviewed and approved by Institutional Animal Care and Use Committee at Yale University.

## Author Contributions

JP, JS, and JD: conceptualization, methodology, writing—review, and editing. JP and JD: software, writing—original draft, and visualization. JP: formal analysis and investigation. JD: supervision. JS and JD: funding acquisition. All authors contributed to the article and approved the submitted version.

## Conflict of Interest

The authors declare that the research was conducted in the absence of any commercial or financial relationships that could be construed as a potential conflict of interest.
