# Supplementary material for: Receptoral Mechanisms for Fast Cholinergic Transmission in Direction-Selective Retinal Circuitry
Source: Front Cell Neurosci. 2020 Nov 26;14:604163. doi: 10.3389/fncel.2020.604163 (PMC7726240; doi:10.3389/fncel.2020.604163)
Supplement: Supplementary file 1 [file Data_Sheet_1.PDF]

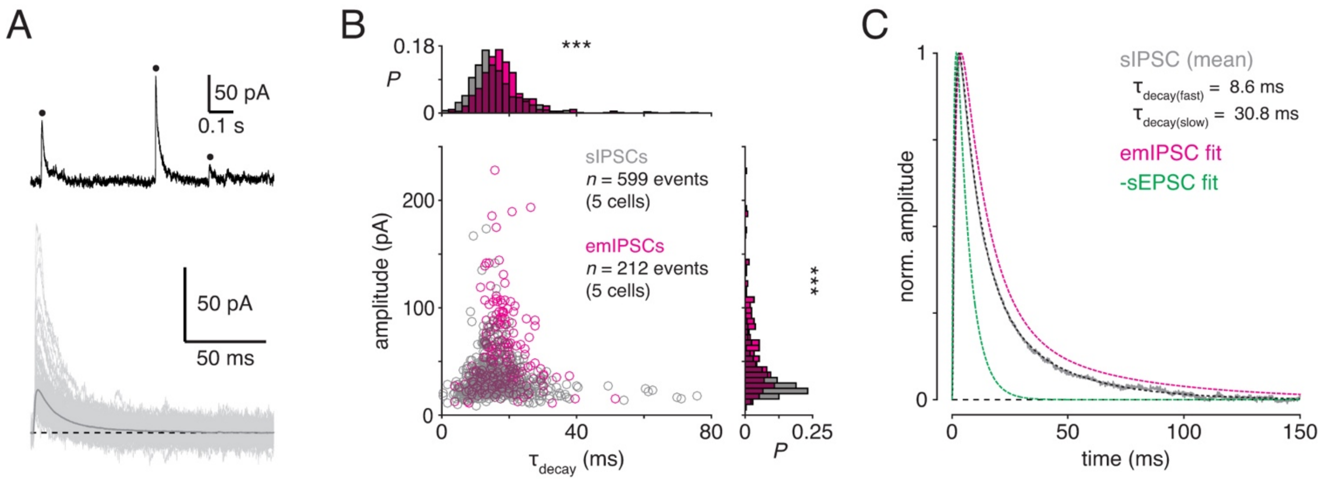


**FIGURE S1** Comparison of spontaneous and evoked monophasic GABAergic IPSCs in DSGCs. **(A)** Isolation and measurement of spontaneous IPSCs (sIPSCs) in DSGCs. *Top*, whole-cell voltage-clamp recording of sIPSCs in an ON-OFF DSGC ($V_{hold}$ ~ 0 mV). Dotted events indicate registered sIPSCs. *Bottom*, 100 individual sIPSCs (light gray) isolated in the same ON-OFF DSGC as above with the mean of all registered events overlaid (dark gray; *n* = 599 events from 4 ON-OFF DSGCs and 1 ON DSGC). **(B)** Comparison of all sIPSCs and emIPSCs recorded in DSGCs. Amplitude is plotted against $\tau_{decay}$ for all recorded sIPSCs (*n* = 599 events) and emIPSCs (*n* = 212 events). Marginal probability distributions of IPSC amplitude and $\tau_{decay}$ are shown at right and above, respectively. IPSC amplitude—evoked vs. spontaneous: ****p* < 0.001; IPSC $\tau_{decay}$—evoked vs. spontaneous: ****p* < 0.001 (Kolmogorov-Smirnov tests). **(C)** Averaged time course of sIPSCs recorded in DSGCs. The average of all sIPSCs recorded in DSGCs (gray) is fit by a function (black, dashed) with two exponential decay terms ($\tau_{decay(fast)}$ = 8.6 ms; $\tau_{decay(slow)}$ = 30.8 ms) and overlaid with fits to the DSGC emIPSC (magenta, dashed) and sEPSC (green, dashed) averages shown in Figure 4D. All traces are normalized to their respective maxima.

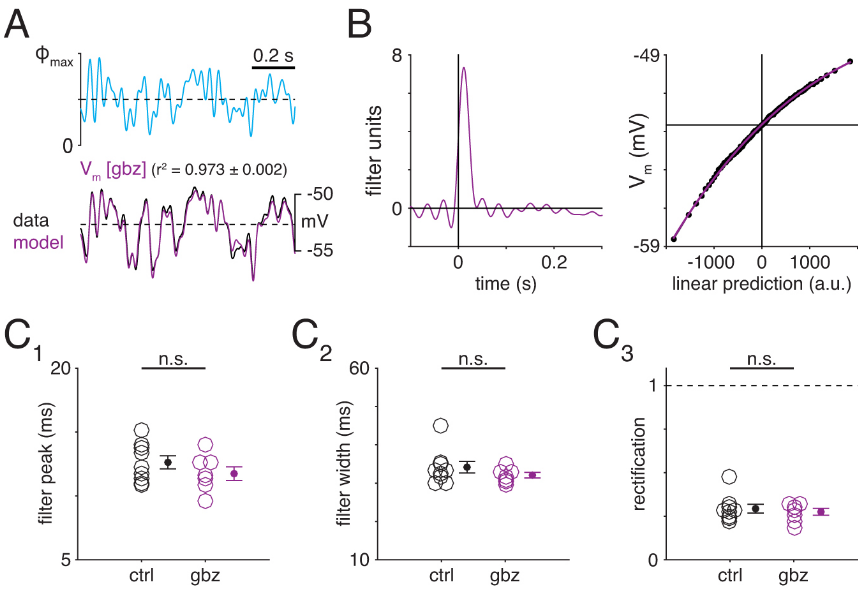


**FIGURE S2** ChR2-modulated voltage responses of SACs during $GABA_A$ receptor blockade. **(A)** Linear-nonlinear model of ChR2-modulated ON SAC $V_m$ during application of gabazine (25 μM). Black trace: mean of 10 responses to repeated stimulus; purple trace: LN model prediction. Maximum light intensity ($\Phi_{max}$) = 3.2 × $10^{17}$ Q $cm^{-2}$ $s^{-1}$. **(B)** Linear filter (*left*) and static nonlinearity (*right*) obtained from a $V_m$ recording in an ON SAC during gabazine application. **(C)** Measurement of LN model components obtained from $V_m$ recordings in ON SACs in the absence (ctrl; *n* = 9 cells) or presence of gabazine (gbz; *n* = 7 cells). Linear filter peak times—ctrl vs. gbz: *p* = 0.287; linear filter widths—ctrl vs. gbz: *p* = 0.240; rectification—ctrl vs. gbz: *p* = 0.480 (two-sample *t*-tests).
